# CD11b^+^ Migratory Dendritic Cells Mediate CD8 T Cell Cross-Priming and Cutaneous Imprinting after Topical Immunization

**DOI:** 10.1371/journal.pone.0091054

**Published:** 2014-03-11

**Authors:** Suzanne T. Nizza, James J. Campbell

**Affiliations:** 1 Department of Dermatology, Brigham and Women's Hospital, Boston, Massachusetts, United States of America; 2 Graduate Program in Biological and Biomedical Sciences, Harvard Medical School, Boston, Massachusetts, United States of America; 3 Department of Dermatology, Harvard Medical School, Boston, Massachusetts, United States of America; University of Pittsburgh, United States of America

## Abstract

Topical antigen application is a focus of current vaccine research. This immunization route mimics natural antigen exposure across a barrier tissue and generates T cells imprinted for skin-selective homing. Soluble antigens introduced through this route require cross-presentation by DC to generate CD8 T cell responses. Here we have explored the relative contribution of various skin-derived DC subsets to cross-priming and skin-selective imprinting. In our model, DC acquire soluble Ag *in vivo* from immunized murine skin for cross-presentation to naïve CD8 T cells *ex vivo*. We find CD11b^+^ migratory DC to be the relevant cross-priming DC in this model. Both Langerin^+^ and Langerin^-^ CD11b^+^ migratory DC can cross-present antigen in our system, but only the Langerin^+^ subset can induce expression of the skin-selective addressin E-selectin ligand. Thus, the CD11b^+^ Langerin^+^ migratory DC population, comprised primarily of Langerhans cells, both cross-primes naïve CD8 T cells and imprints them with skin-homing capabilities.

## Introduction

Dendritic cells (DC) are the primary antigen (Ag)-presenting cells that initiate T cell responses. DC were first recognized for presenting exogenous peptides to CD4 T cells via MHCII, and endogenous peptides to CD8 T cells via MHCI (reviewed in [1]). Cross-presentation, a more recently recognized DC function, is the presentation of exogenous peptides to CD8 T cells via MHCI (reviewed in [2]). Cross-presentation is required to initiate responses against tumor cells and intracellular pathogens that do not directly infect DC.

DC subsets can be distinguished by surface markers and by their positioning within tissues. DC within the skin-draining lymph nodes (sdLN) include plasmacytoid DC, LN-resident (classical) DC and migratory DC, which migrate from skin to sdLN. Classical DC are MHCII^int^CD11c^hi^ and contain CD8α^+^CD11b^-^ and CD8α^-^CD11b^+^ subsets [3]. Migratory DC can transport Ag from skin to sdLN via afferent lymphatics [4], [5], are MHCII^hi^CD11c^int^, and require functional CCR7 expression to enter sdLN. This population contains CD11b^-^CD103^+^ and CD11b^+^CD103^-^ subsets. Langerin (CD207) is expressed by all epidermal migratory DC (*i.e.* Langerhans cells, LC) and some dermal migratory DC [6], [7]. Despite common Langerin expression, LC and Lang^+^ dermal DC are functionally and developmentally distinct subsets [8].

CD8α^+^ classical DC are considered the primary subset for cross-priming naïve CD8 T cells [2], [9] and may possess specialized intracellular machinery for processing and presenting exogenous Ag on MHCI [10]. Recent studies suggest that CD103^+^ migratory DC also cross-present Ag [11]. However, some of these studies used viruses that may directly infect some DC [12], so these findings may be attributable to classical MHCI presentation of endogenous Ag.

DC can imprint naïve T cells to express homing molecules that direct the primed T cells to preferentially enter certain barrier tissues, as reviewed in [13], [14]. For example, T cells in peripheral blood use the carbohydrate ligand of E-selectin (E-lig, or CLA in humans) to enter skin and integrin α4β7 to enter intestinal tissues [13]. Prior work showed that peptide-pulsed DC from sdLN or Peyer's patches can imprint CD8 T cells to express E-lig or α4β7, respectively [15].

We set out to more clearly define the *in vivo* DC subsets that cross-present cutaneous soluble Ag and/or imprint naïve CD8 T cells with skin-homing profiles. We used a murine *in vivo* system in which DC acquire Ag from inflamed skin. We isolated these “Ag-charged” DC from the sdLN of immunized mice and tested their ability to cross-prime Ag-specific naïve CD8 T cells *ex vivo*. This allowed us to test the contribution of various DC subsets to cross-priming and tissue-selective imprinting.

## Materials and Methods

### Mice

C57Bl/6 CD45.2 mice were purchased from Charles River Labs (Wilmington, MA). Lang-DTREGFP (Lang-DTR) and Lang-EGFP mice were a generous gift from Bernard Malissen, Centre d'immunologie de Marseille Luminy [16]. CCR7^-/-^ [17] and CD45.1 OT-I [18] mice were from our colony, the founders obtained from Jackson Labs. (TCRα^-/-^ OT-I T cells were used for some experiments, but no differences in proliferation were seen with respect to TCRα^+^ OT-I T cells.)

### Topical Skin Immunization

Topical immunization of ear skin was performed as described in [19], [20]. Briefly, the stratum corneum on each side of each ear was gently stripped with ten applications of adhesive tape (Scotch matte finish magic tape, 3 M), taking care not to break the skin or cause bleeding. To remove cutaneous lipids that would repel Ag in aqueous solution, 25 µl of acetone was spread over each ear. After evaporation of the acetone, 25 µl of an aqueous mixture containing 1 mg/ml cholera toxin (CT) adjuvant (List Biological Labs, Campbell, CA) was applied to each ear and uniformly spread with a small paint brush. Control mice received only the CT adjuvant, while experimental mice also received 50 µl of an aqueous mixture containing 100 mg/ml ovalbumin (OVA) Ag (Sigma-Aldrich product #A5503) on each ear.

### Treatment of Lang-DTR mice with Diphtheria Toxin

For some cocultures (as indicated), WT or Lang-DTR mice were treated with diphtheria toxin (DT) (List Biological Labs, Campbell, CA). Mice were injected intraperitoneally with 1 µg DT in 100 µl PBS. Mice were treated one day preceding immunization and one day after immunization.

### Dendritic Cell Isolation

SdLN were harvested from immunized mice, then disrupted between frosted microscope slides and filtered through 80 µm mesh. Remaining solid stroma was incubated for 30 min at 37 C with 1 mg/ml collagenase (Sigma). Digested product was filtered through mesh and added to the rest of the LN prep prior to washing and counting.

### Dendritic Cell Sorting

DC were enriched from the sdLN prep after exclusion of T and B cells with anti-B220 (clone RA3.3A1/6.1, ATCC hybridoma supt) and mThy-1.2 Ab (BioXCell, West Lebanon, NH) followed by incubation with mαr Igκ microbeads (Miltenyi Biotec, Auburn, CA) and sorting on an AutoMACS Separator. CD8α^+^ DC selection was done using the CD8^+^ dendritic cell isolation kit, mouse (Miltenyi). Isolation of individual DC subsets was performed on a BD FACSARIA (Becton Dickinson, San Jose, CA).

### T Cell Preparation

Spleens were harvested from CD45.1 OT-I (or OT-I TCRα^-/-^) mice for single cell suspensions. RBC were lysed and remaining cells washed and loaded with CFSE. CD8^+^ T cell selection was performed using “CD8^+^ T cell isolation kit II, mouse” (Miltenyi).

### Coculture

After sorting, isolated populations were resuspended in 5 ml RPMI+10% FBS and a sample taken to identify DC or CD8 T cells by FACS analysis. A known number of 5 µm beads was added for accurate counting. DC and T cells were plated for co-culture in 96-well round-bottom plates. A 1∶1 DC:T cell ratio was found to provide the most reproducible T cell proliferation and was maintained for all co-cultures. This high ratio was likely required due to the relative rarity of DC carrying *in vivo*-acquired Ag. Co-cultures were incubated for 6 days and cells stained for FACS analysis.

### *In vivo* Transfers

CD45.1 OT-I spleen and pLN were harvested and single cell suspensions prepared. Red blood cells were lysed and remaining cells were washed and loaded with CFSE. After counting, approximately 1.5×10^7^ T cells were retro-orbitally injected into anesthetized mice. Mice were immunized on ear skin (as described above) and LNs were harvested and analyzed for T cell proliferation five days later. WT and Lang-DTR mice were used as recipients. DT-treated mice were injected with DT one day before and one day after T cell transfer. Timeline: day -2, first DTX treatment; day -1, OT-I cells transferred IV to recipients; day 0, ear skin immunized and second DTX treatment given; day 5, skin-draining LN harvested.

### Flow Cytometry

Directly conjugated mAbs were purchased from eBioscience (La Jolla, CA) or BD Pharmingen (San Jose, CA). Flow cytometry was performed on a BD FACS Canto (Becton Dickinson) and analyzed by FlowJo software version 8.8.6 (Treestar, Inc., Stanford, CA).

### Statistics

All statistics were performed using one-tailed Mann-Whitney *U*-tests using Prism software version 5.0a (GraphPad, Inc., La Jolla, CA).

### Ethics Statement

This study was carried out in strict accordance with the recommendations in the Guide for the Care and Use of Laboratory Animals of the National Institutes of Health. The protocol was approved by the Institutional Animal Care and Use Committee of Harvard Medical School (Animal Welfare Assurance of Compliance number: A3431-01). All immunizations were performed under ketamine and xylazine anesthesia and all efforts were made to minimize suffering.

## Results

### Measuring functionality of individual DC subsets from skin

We set out to examine CD8 T cell cross-priming after the cutaneous introduction of soluble Ag. Our approach involves two stages, the first *in vivo* and the second *ex vivo* (Fig S1). C57Bl/6 wildtype (WT) mice (or genetically-modified mice on the C57Bl/6 background) were immunized with OVA protein on ear skin along with cholera toxin (CT) adjuvant. CT was chosen because its properties as an adjuvant suggest that it is a promising candidate for topical vaccination of human patients [21]. After immunization, “Ag-charged” DC were isolated from cervical LN, which are a primary sdLN downstream of the ear skin. At the same time, splenic CD8 T cells were isolated from naive OT-I mice, which express a transgenic TCR specific for the H2-K^b^-restricted peptide OVA_257-264_. The DC-enriched sdLN cells and OT-I T cells were then co-cultured *ex vivo*.

**Figure 1:**
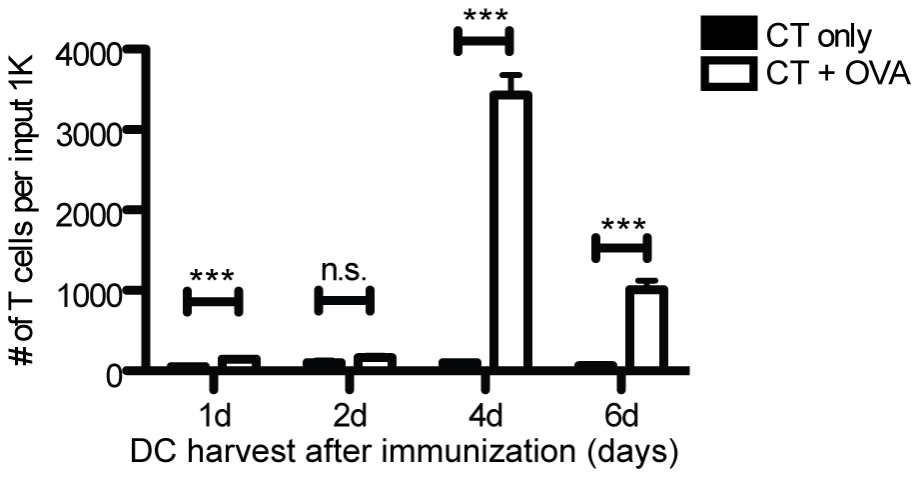
Time course for peak day of sdLN DC harvest after topical immunization for OT-I proliferation. DCs harvested from sdLN various days after topical ear skin immunization and co-cultured with CFSE-labeled naïve OT-I cells. *Black bars*: CT (adjuvant) only. *White bars*: CT + OVA protein. CD45.1^+^ CD3^+^ CD8^+^ cells were gated for assessment of CFSE loss. sdLN from 3-4 mice per condition were pooled before DC isolation. Results are from two (1d, 6d) or three (2d, 4d) independent experiments. Proliferation measured at d6 of *ex vivo* culture. One-tailed Mann-Whitney *p* values shown. *p<.05; ***p<.0001; n.s.  =  not significant.

The sole source of Ag in our experimental co-cultures is the OVA protein that was topically applied several days earlier to the DC donor mice. We also established control co-cultures in which exogenous OVA was added directly to culture wells, to confirm DC viability and functionality. Fig 1 shows that DC from sdLN isolated four days post-immunization yielded the maximal OT-I proliferative response.

### The migratory DC subset is essential for CD8 T cell proliferation

**Figure 2:**
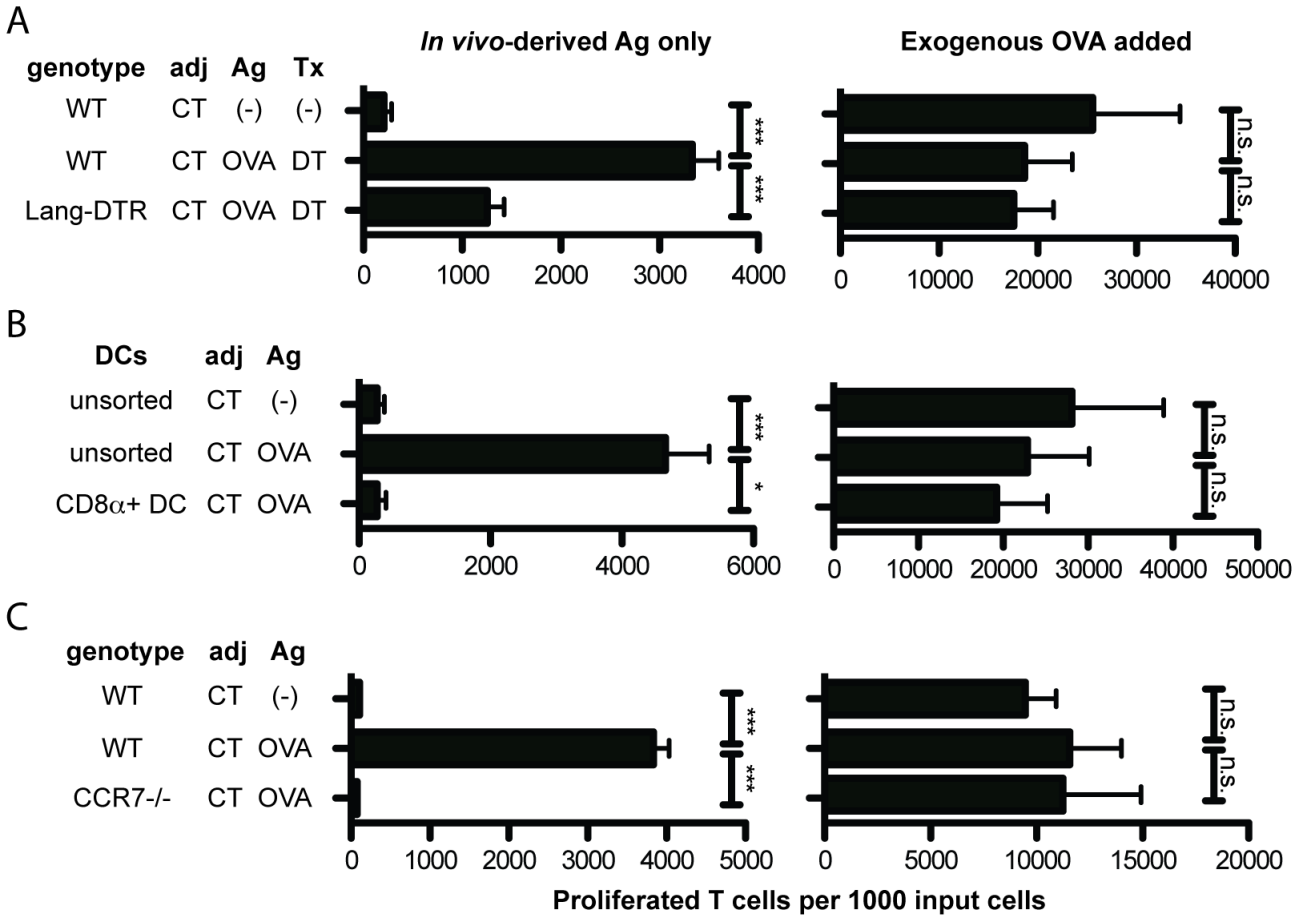
Migratory DCs are essential for CD8^+^ T cell proliferation after topical immunization. ***A***: WT and Lang-DTR mice were topically immunized with CT + OVA protein or CT alone. Mice were injected with diphtheria toxin (DT) five and three days prior to cell isolation. On day 4 post-immunization, sdLN cells were isolated, enriched for DCs, and co-cultured with CFSE-labeled OT-I T cells. sdLN from 4–5 mice per condition were pooled before DC isolation. N = 4 experiments. ***B***: CD8α^+^ DCs were isolated from immunized WT mice using an AutoMACS kit. Unsorted DCs were treated as in *A*. DCs were cultured with CFSE-labeled OT-I T cells. sdLN from 4–5 mice per condition were pooled before DC isolation. N = 3 experiments. ***C***: WT and CCR7^-/-^ mice were immunized and DC harvested on d4 post-immunization and cultured with CFSE-labeled OT-I cells. sdLN from 4–5 mice per condition pooled before DC isolation. N = 3 experiments. For all experiments shown, T cell proliferation was analyzed on day 6 of *ex vivo* culture. Proliferated T cells per 1000 input T cells is depicted. Flow cytometry plots were gated on CD45.1^+^ CD3^+^ CD8^+^ cells. Adj  =  adjuvant. Ag  =  antigen. Tx  =  treatment. *Left panels*: The only Ag present in co-culture wells was that carried by DC from immunized mice. *Right panels*: Exogenous OVA protein was directly added to “positive control” wells. One-tailed Mann-Whitney *p* values shown. *p<.01; ***p<.0001; n.s.  =  not significant.

To determine whether Lang-expressing DC are necessary for cross-presentation, we used Lang-DTR-EGFP (Lang-DTR) mice. Diphtheria toxin (DT) receptor is knocked into the Langerin locus, and treatment with DT leads to selective loss of Lang^+^ DC, including LC, within 24 hours [7]. WT or Lang-DTR mice were treated with DT and immunized on ear skin. We found Lang-depleted DC to be only ∼50% as efficient as WT DC at stimulating OT-I proliferation in our 1∶1 DC/T cell co-cultures (Fig 2A, left). The defect was restricted to Ag acquired *in vivo*, as both populations were able to cross-present exogenous Ag added to the culture wells *ex vivo* (Fig 2A, right).

We next focused on the CD8α^+^ DC subset. Interestingly, the capability to cross-present *in vivo*-acquired Ag did not reside within this population (Fig 2B, left). CD8α^+^ DCs could, however, cross-present exogenous OVA (Fig 2B, right).

We next assessed the cross-presentation ability of classical DC in the absence of migratory DC. CCR7^-/-^ mice lack migratory DC in sdLN, as CCR7 is required for DC migration from skin [17]. SdLN DC from CCR7^-/-^ mice were unable to cross-prime OT-I cells (Fig 2C, left). CCR7^-/-^ sdLN DC could cross-present exogenous OVA (Fig 2C, right).

These data, if considered by themselves, suggest that migratory DC are required in the LN for direct Ag presentation to T cells *or* to “license” CD8α^+^ DC [2]. To distinguish between these possibilities, we asked whether migratory DC from sdLN could independently cross-present Ag acquired *in vivo*.

### The ability to cross-prime naïve CD8 T cells with *in vivo*-acquired Ag resides within the migratory DC subset

**Figure 3:**
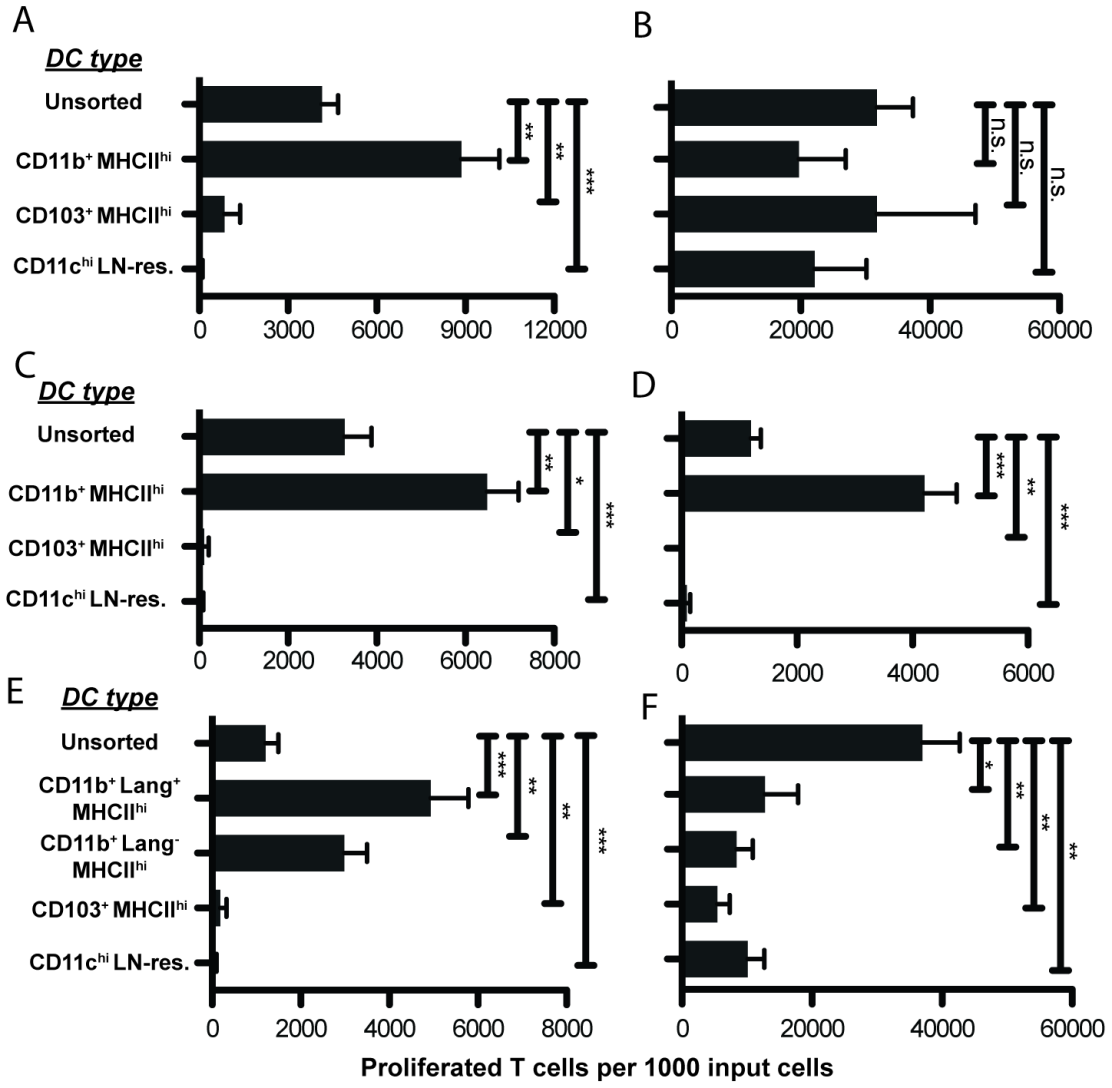
Cross-priming ability resides within the CD11b^+^ MHCII^hi^ subset. ***A***: Sorted DCs from immunized mice co-cultured with CFSE-labeled OT-I T cells. The only Ag present in co-culture wells was that carried by DC from immunized mice. Mice were immunized four days before lymph node harvest. N = 3 experiments. ***B***: “Positive control” for *3A* - exogenous OVA protein added to wells to confirm DC viability and functionality post-sorting. N = 3 experiments. ***C***: Mice were immunized on ear skin four days before lymph node harvest as previously described, except LPS was used as adjuvant instead of cholera toxin. DCs were sorted and cultured as in *3A*. N = 3 experiments. ***D***: Sorted DCs from immunized mice co-cultured with CFSE-labeled OT-I T cells. Mice were immunized **two** days before lymph node harvest, instead of 4 days. N = 2 experiments. ***E***: CD11b^+^ migratory DCs were subdivided into Lang^+^ and Lang^-^ subsets. Sorted DCs from mice immunized four days before lymph node harvest were co-cultured with CFSE-labeled OT-I T cells. N = 4 experiments. ***F***: “Positive control” for 3*E*. N = 4 experiments. For all experiments shown, sdLN from 10 immunized mice were pooled before DC sorting. T cell proliferation was analyzed on d6 of *ex vivo* culture with sdLN DC isolated on d4 after immunization (except for 3*D*). The number of proliferated T cells per 1000 input T cells is depicted. Flow cytometry plots were gated on CD45.1^+^CD3^+^CD8^+^ cells. One-tailed Mann-Whitney *p* values shown. *p<.05; **p<.001; ***p<.0001; n.s.  =  not significant.

We used fluorescent cell sorting to separate the MHCII^hi^CD11c^int^ migratory DCs into CD11b^+^ CD103^-^ and CD11b^-^ CD103^+^ subsets (Fig S2A). We also isolated MHCII^int^CD11c^hi^ classical DC (which contain the CD8α^+^ DC population) for comparison with migratory DC. Surprisingly, we found that CD11b^+^ migratory DC were the only subset to induce appreciable T cell proliferation in co-culture; CD103^+^ DC and classical DC stimulated negligible proliferation (Fig 3A). All DC populations could cross-present exogenous OVA (Fig 3B), proving them to remain viable and functional after sorting.

This unexpected finding was not specific to the CT adjuvant, as CD11b^+^ migratory DC remained the sole cross-presenting population when LPS was used as an alternative adjuvant (Fig 3C). Furthermore, this finding was not specific to the time point chosen for harvesting DC after immunization (day 4); we performed identical experiments at day 2 and found that CD11b^+^ migratory DC remained the only subset to induce appreciable T cell proliferation in co-culture (albeit the proliferation was somewhat less at this earlier time point) (Fig 3D).

We next used Lang-EGFP mice [16] as the DC source to allow subdivision of the CD11b^+^ migratory DC population into Lang^+^ and Lang^-^ subsets by fluorescent sorting (Fig S2B). Both populations stimulated significant T cell proliferation (Fig 3E). Again, all DC populations could cross-present exogenous OVA added to the culture wells (Fig 3F), proving them to remain viable and functional after sorting.

### CD11b^+^ Langerin^+^ DC are responsible for the majority of E-lig imprinting on T cells

DC are capable of imprinting primed T cells to express tissue-selective homing markers [13], [14], [22]. E-lig is required for entry of T cells into skin from blood [13]. Early work showed that peptide-pulsed DC from sdLN could stimulate E-lig expression on CD8 T cells [15], but direct exposure of DC to peptide Ag *in vitro* does not maintain key components of *in vivo* Ag transport and processing.

**Figure 4:**
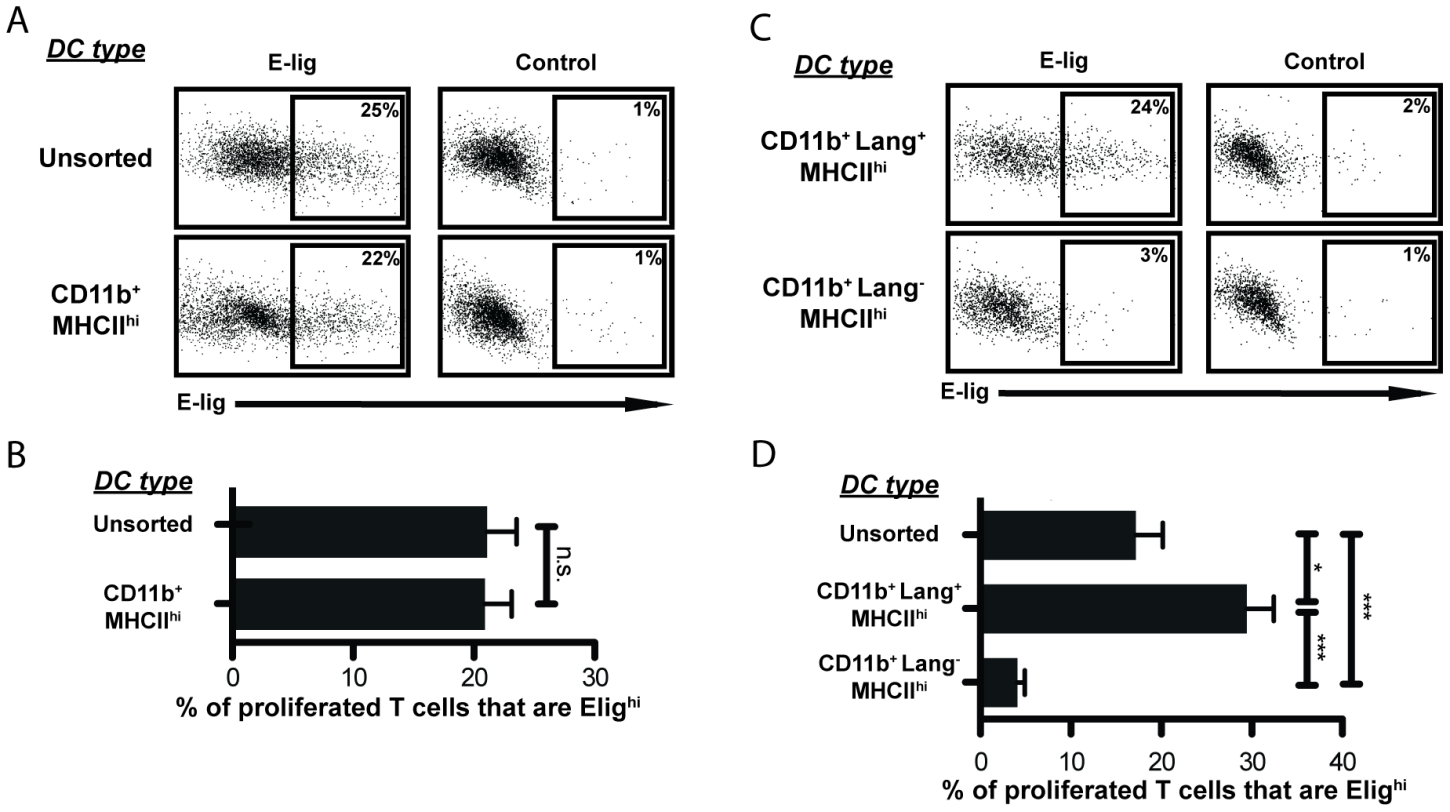
CD11b^+^Langerin^+^MHCII^hi^ DCs induce E-lig. ***A***: Representative example of E-lig expression on CD8 T cells after co-culture with unsorted DCs or sorted CD11b^+^ MHCII^hi^ DCs. Control was stained in the presence of EDTA (which disrupts Ca^++^-dependent E-selectin binding) to determine non-specific binding of the E-selectin-Ig chimera. Flow cytometry plots are gated on CFSE-low CD8^+^ T cells. Vertical axis: side scatter. Plots representative of 3 different experiments of 5–8 wells each (depending on DC yield). 3433 cells shown per panel. ***B***: E-lig expression depicted as the percentage of proliferated T cells expressing high levels of E-lig. E-lig^hi^ expression was observed only on proliferated T cells, so undivided T cells are not included in the calculation. Data from co-cultures with unsorted DCs or sorted CD11b^+^ MHCII^hi^ DCs are compared within a given experiment. sdLN from 10 immunized mice were pooled before DC sorting. N = 3 experiments. One-tailed Mann-Whitney *p* values shown. n.s.  =  not significant. ***C***: Representative example of E-lig expression on CD8 T cells after co-culture with CD11b^+^ Lang^+^ or CD11b^+^ Lang^-^ MHCII^hi^ DCs. Control stained as in *A*. Flow cytometry plots are gated on CFSE-low CD8^+^ T cells. Vertical axis: side scatter. Plots are representative of samples from 4 different experiments of 3–8 wells each (depending on DC yield). 1623 cells shown per panel. ***D***: E-lig expression depicted as the percentage of proliferated OT-I T cells that are E-lig^hi^ as in *B*, Data from co-cultures with unsorted DCs and sorted Lang^+^ or Lang^-^ CD11b^+^ MHCII^hi^ DCs compared within a given experiment. sdLN from 10 immunized mice were pooled before DC sorting. N = 4 experiments. One-tailed Mann-Whitney *p* values shown. *p<.01; ***p<.0001.

Unsorted DC induced E-lig expression on proliferating CD8 T cells, and sorted CD11b^+^ migratory DC retained this function (Fig 4A, B). However, after sorting the Lang^+^ and Lang^-^ subsets, we found that the ability to induce E-lig expression resided largely within the Lang^+^ population (Fig 4C, D). This is informative, as previous work suggested that activated CD8 T cells express E-lig by default unless they receive gut-specific imprinting signals [15], [23].

### *In vivo* verification of *ex vivo* findings

**Figure 5:**
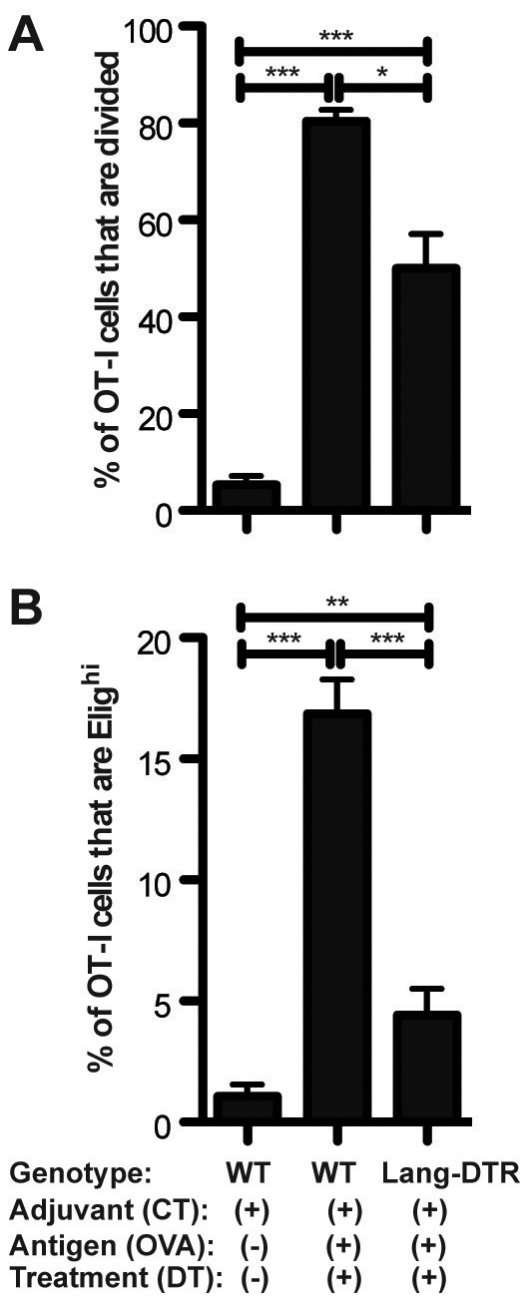
Decreased proliferation and E-lig induction for OT-I cells adoptively transferred into Lang^+^ DC-depleted mice. CFSE-labeled splenocytes from CD45.1+ OT-I donor mice were injected *iv* into recipient WT and Lang-DTR mice. On the following day, recipients were topically immunized with CT+OVA protein or CT alone on the ear skin, using the same immunization techniques as in our *in vivo/ex vivo* assays. All mice were treated with twice diphtheria toxin. *Timeline*: day -2, first DTX treatment; day -1, OT-I cells transferred IV to recipients; day 0, ear skin immunized and second DTX treatment given; day 5, skin-draining LN harvested. ***A***: Proliferation depicted as the percentage of total OT-I T cells that are CFSE low. ***B***: E-lig expression depicted as the percentage of total. N = 3 experiments of 4–5 mice per group. For all experiments shown, sdLN cells were isolated and gated on CD45.1+CD3+CD8+ cells. One-tailed Mann-Whitney *p* values shown. *p<.05; **p<.001; ***p<.0001; n.s.  =  not significant.

Our *in vivo/ex vivo* assay suggested that CD11b^+^Lang^+^ DC are the key subset mediating E-lig induction on naïve CD8 T cells after topical immunization. This result implied that a mouse lacking Lang^+^ DC would be impaired in its ability to produce E-lig^+^ T cells after topical immunization. To test this hypothesis, naïve OT-I cells were transferred directly into DTX-treated Lang-DTR mice for comparison to other recipient mice bearing normal numbers of Lang^+^ DC. In the absence of Lang^+^ DC, we found the induction of E-lig expression on OT-I cells to be reduced dramatically (Fig 5).

## Discussion

There are at least three general mechanisms through which DC can obtain cutaneous Ag from skin: *1*) migratory DC transport Ag directly to the sdLN [24]; *2*) LN-resident classical DC obtain soluble Ag within the sdLN from afferent lymph [25]; or *3*) LN-resident classical DC obtain Ag within the sdLN by transfer from migratory DC [4]. The third mechanism is currently believed to generate effective cross-presentation *in vivo* (reviewed in [2]).

It is important to understand the mechanism by which immunization occurs through injured skin as investigated in this study. One of the most successful immunization procedures in history is Edward Jenner's 18^th^ century inoculation of patients with non-virulent vaccinia virus through skin scarification to protect against smallpox. It has been proven today that the skin scarification method of immunization is many times more effective for this particular virus than more modern inoculation methods such as subcutaneous injection [26]. This suggests that antigen delivery through skin can induce a type of immune reaction different from those induced through other routes.

We have established a model system to study cross-presentation by loading DC with Ag through a tissue-specific immunization route *in vivo*, and used these “Ag-charged” DC to elicit proliferation and activation of Ag-specific naïve T cells *ex vivo*. Our approach goes beyond the abilities of many existing models, which remove any physical or mechanistic barriers that might normally prevent a given DC subtype from accessing Ag *in vivo*. Our approach preserves the loading of DC with Ag within the complex three-dimensional developmental niches of the intact skin and sdLN, which would be difficult if not impossible to accurately reproduce *in vitro* with current technology. Thus, our approach combines the robustness of a reductionist *ex vivo* model with the crucial *in vivo* components of Ag-acquisition by DC and subsequent transport of Ag to the LN. This approach has provided us with significant advantages towards understanding the cells, molecules and processes required for Ag cross-presentation, and the potential for such immunization routes to generate tissue-specific cellular immune responses in human patients.

The question of which skin-resident DC subset is responsible for the cross-presentation of soluble Ag is quite well studied, but far from resolved. Our results demonstrate that CD11b^+^ migratory DC cross-present soluble protein Ag applied to skin. We found all other DC subsets essentially incapable of cross-priming CD8 T cells with *in vivo*-acquired Ag. This is surprising, given the association of CD8α^+^ classical DCs and CD103^+^ migratory DCs with cross-presentation in other contexts, especially as these DCs are thought to be developmentally related [2], [9], [11]. However, under our co-culture conditions, neither of these subsets was able to cross-prime CD8 T cells. We found that CD11b^+^ migratory DC cross-prime CD8 T cells directly, not by licensing other DC populations. By using an approach that requires DC to acquire Ag *in vivo*, we have preserved the physical barriers that may prevent some DC from accessing Ag *in vivo*. As all DC subsets tested were capable of cross-presenting Ag added directly to culture wells, this finding emphasizes the importance of Ag access, which can only be observed *in vivo*.

Most interestingly, we also found that not all DC subsets that can cross-prime CD8 T cells are also capable of imprinting them for skin-selective homing (Fig 4). Our data demonstrate that the entire population of CD11b^+^ migratory DC can cross-present *in vivo*-acquired Ag, but only the Lang^+^ subset could effectively imprint the activated CD8 T cells to express E-lig. This finding was confirmed *in vivo* by demonstrating that adoptively transferred OT-I populations within the skin-draining nodes of hosts lacking Lang^+^ DC contained dramatically fewer E-lig^+^ cells.

Some have suggested that imprinting T cells to express homing markers is independent of activation site or tissue of origin [27]. Other work has focused on stromal or LN microenvironment factors [28], [29]. The variation in E-lig imprinting between Lang^+^ and Lang^-^ CD11b^+^ migratory DC strongly suggests that skin-selective imprinting requires signals distinct from those that stimulate proliferation. It also implies that imprinting is an instructive process orchestrated by DC, independent of contemporaneous exposure to afferent lymph or LN-derived stroma cells (albeit such factors may influence DC imprinting capabilities prior to *ex vivo* culture).

The immunophenotype of the population we found capable of both cross-presentation and imprinting is consistent with that of LC (*i.e.* Lang^+^ CD11b^+^ CD103^-^ DC within the MHCII^hi^ CD11c^int^ migratory DC population). The cross-presentation capability of LC has been controversial, with some showing LC to be unnecessary for CD8 T cell responses [30], [31], and others showing them to be important [21], [32]. Work with LC ablation models has offered mixed results with regard to the role of LC in contact hypersensitivity (reviewed in [33]). The finding that LC transfer HSV Ag to CD8α^+^ DC that then prime CD8 T cells supports the idea that LC do not directly cross-prime T cells [4]. However, other work has found LC pulsed *ex vivo* with Ag to be capable of cross-presentation [34], [35]. The present study demonstrates that each DC subset we isolated from sdLN is *capable* of cross-presentation when pulsed with OVA *ex vivo* (Figs 3B, 3F). Importantly, however, we have demonstrated that when OVA is applied physiologically, requiring passage of Ag through the skin barrier tissue, only the CD11b^+^ migratory DCs are able to cross-present. While the LCs are not the only DC subset to cross-present (the Lang^-^ CD11b^+^ migratory DCs do so as well), they are the only DC subset able to imprint E-lig.

The lack of consensus on LC cross-presentation ability may stem from the use of different Ag under varying immunization conditions. DC subsets that do not typically cross-present Ag may do so when competing DCs are removed. Using LC depletion models to examine the role of LC could thus obscure their true role *in vivo*, especially if the depletion is chronic. However, acute depletion models are useful for narrowing down DC subsets for further investigation, as we have done in the current study. In addition, it is possible that LC directly present some Ag (*e.g.* soluble protein Ag), but transfer others (*e.g.* HSV Ag) to LN-resident DC. Further studies are required to build a complete picture for the role of LC in cross-presentation, but we have demonstrated the ability of LCs to both cross-present and imprint skin-homing markers on CD8 T cells.

Interestingly, LC are often considered to be tolerogenic, as LC internalize self-Ag in the steady state without causing autoimmune responses [36]. LC also arrive late in the sdLN and inefficiently induce CD80 and CD86 [24]. However, it should be noted that peak T cell proliferation occurred in our system when DC were harvested at day 4 after *in vivo* immunization (Fig 1), the peak day for LC arrival in the sdLN [24]. It is possible that LC tolerogenicity depends on maturation state (reviewed in [37]), as immature LC can migrate to draining LN [38]. Also, as LC were not distinguished from Lang^+^ dermal DC in many earlier studies, Lang^+^ dermal DC may cause the tolerogenic effects currently ascribed to LC [11], [39], reviewed in [37].

We found that the ability to cross-present skin-derived exogenous protein Ag under our experimental conditions resided within the CD11b^+^ subset of CCR7^+^ migratory DC. Within this subset, only the Lang^+^ DCs efficiently induced E-lig expression. This is a novel biological finding, supporting the notion that tissue-selective imprinting is an instructive process. It would be enlightening to further explore how different environmental conditions affect the DC's ability to cross-present; this would be key for DC vaccine design, as targeting a vaccine to a specific DC subset would only be useful if that subset cross-presented Ag under the conditions used for vaccine application.

## Supporting Information

Figure S1 Description of cell isolation and co-culture setup. DCs: cells were harvested from skin-draining lymph nodes (sdLN) post-topical skin immunization on ears and pooled within each group. Pooled cells were depleted of T and B cells and counted. T cells: spleen from OT-I mouse was harvested and red blood cells were lysed. Cells were labeled with CFSE and enriched for CD8^+^ T cells with an AutoMACS kit. DCs and T cells were cultured in a 1∶1 ratio. After 6 days of culture, cells were harvested and stained for flow cytometry analysis.
(TIF)

Figure S2 Depiction of dendritic cell subset sorting strategies. ***A***: To isolate DCs for Fig *3A*, sdLN cells were first sorted on MHCII vs CD11c expression. CD11c^hi^ MHCII^int^ (“LN-resident”) dendritic cells were sorted as one population. CD11c^int^ MHCII^hi^ migratory DCs were sorted into CD11b^+^ and CD103^+^ sub-populations. ***B***: To isolate DCs for Fig *3D*, sdLN were first sorted based on MHCII vs CD11c expression. CD11c^hi^ MHCII^int^ (“LN-resident”) dendritic cells were sorted as one population. CD11c^int^ MHCII^hi^ migratory DCs were sorted into CD11b^+^ and CD103^+^ sub-populations; CD11b^+^ DCs were further divided based on Langerin-EGFP expression.
(TIF)
