# Supplementary figures and images for: CD11b+ Migratory Dendritic Cells Mediate CD8 T Cell Cross-Priming and Cutaneous Imprinting after Topical Immunization
Source: PLoS One. 2014 Mar 11;9(3):e91054. doi: 10.1371/journal.pone.0091054 (PMC3949685; doi:10.1371/journal.pone.0091054)

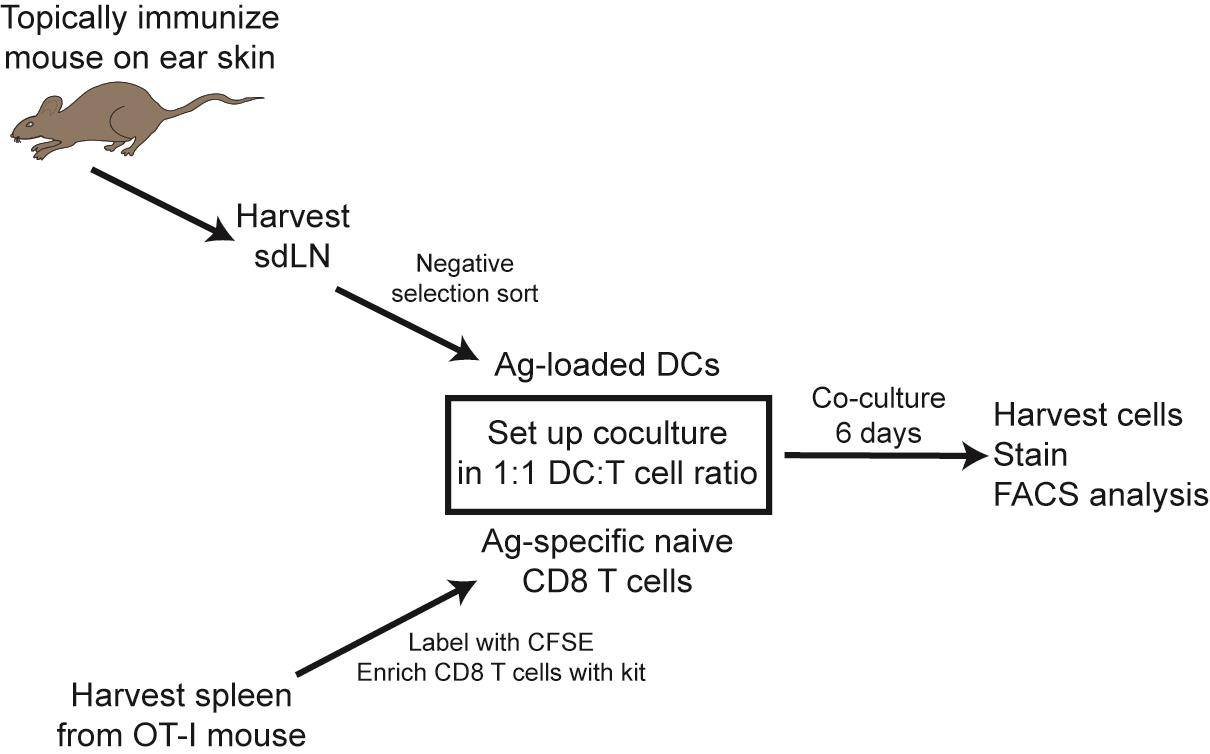

Supplement: Figure S1 — Description of cell isolation and co-culture setup. DCs: cells were harvested from skin-draining lymph nodes (sdLN) post-topical skin immunization on ears and pooled within each group. Pooled cells were depleted of T and B cells and counted. T cells: spleen from OT-I mouse was harvested and red blood cells were lysed. Cells were labeled with CFSE and enriched for CD8+ T cells with an AutoMACS kit. DCs and T cells were cultured in a 1∶1 ratio. After 6 days of culture, cells were harvested and stained for flow cytometry analysis. (TIF) [file pone.0091054.s001.tif]

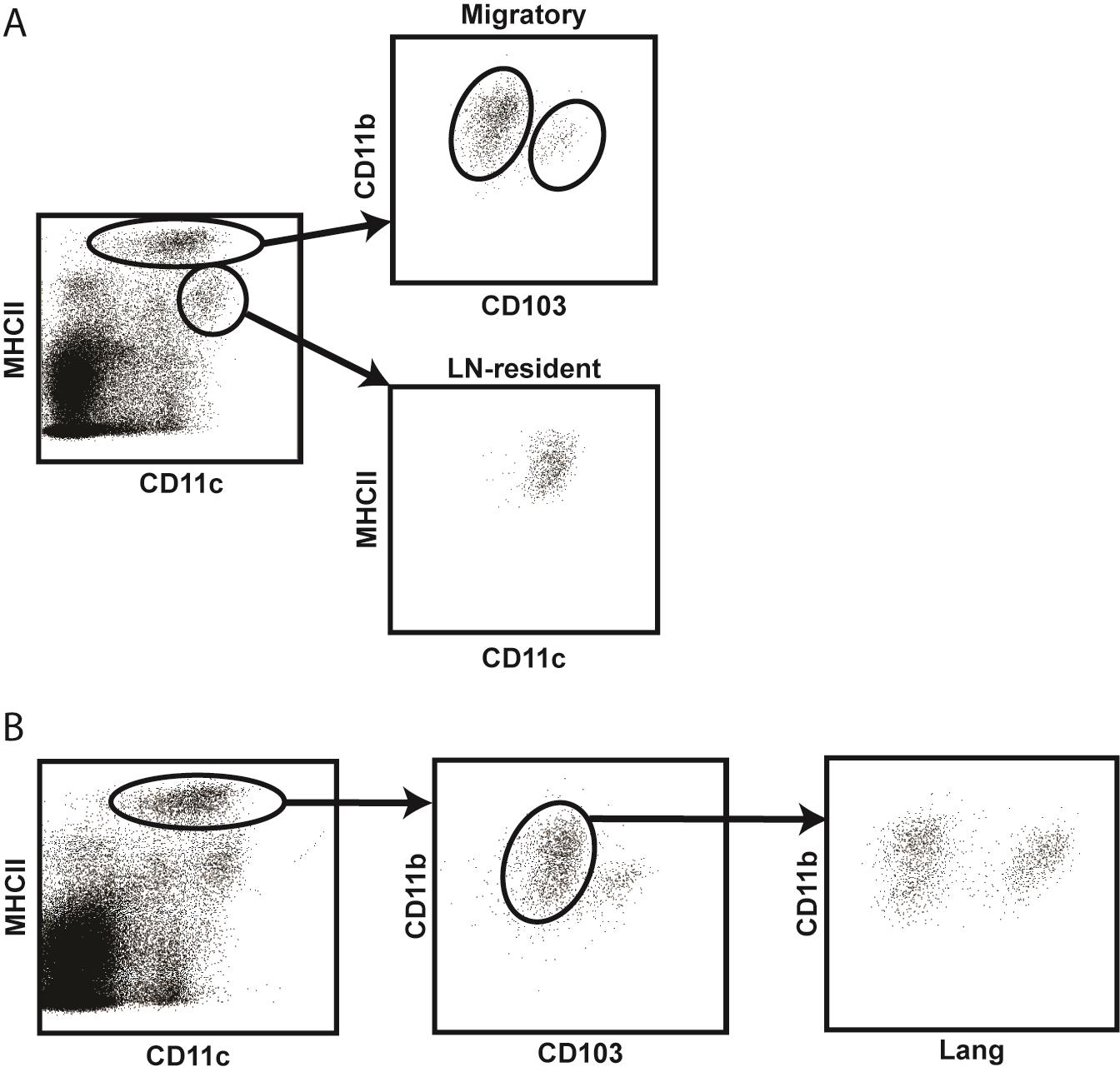

Supplement: Figure S2 — Depiction of dendritic cell subset sorting strategies. A: To isolate DCs for Fig 3A , sdLN cells were first sorted on MHCII vs CD11c expression. CD11chi MHCIIint (“LN-resident”) dendritic cells were sorted as one population. CD11cint MHCIIhi migratory DCs were sorted into CD11b+ and CD103+ sub-populations. B: To isolate DCs for Fig 3D , sdLN were first sorted based on MHCII vs CD11c expression. CD11chi MHCIIint (“LN-resident”) dendritic cells were sorted as one population. CD11cint MHCIIhi migratory DCs were sorted into CD11b+ and CD103+ sub-populations; CD11b+ DCs were further divided based on Langerin-EGFP expression. (TIF) [file pone.0091054.s002.tif]
